# An updated examination of the perception of barriers for pharmacogenomics implementation and the usefulness of drug/gene pairs in Latin America and the Caribbean

**DOI:** 10.3389/fphar.2023.1175737

**Published:** 2023-05-11

**Authors:** Aimeé Salas-Hernández, Macarena Galleguillos, Matías Carrasco, Andrés López-Cortés, María Ana Redal, Dora Fonseca-Mendoza, Patricia Esperón, Farith González-Martínez, Ismael Lares-Asseff, Alberto Lazarowski, Verónica Loera-Castañeda, Diadelis Remírez, Matías F. Martínez, Rodrigo Vargas, Fabricio Rios-Santos, Antonio Macho, Juan P. Cayún, Germán R. Perez, Carolina Gutierrez, Leslie C. Cerpa, Tamara Leiva, Susan Calfunao, Lesly Xajil, Christopher Sandoval, Marcelo Suárez, Ariana Gonzalez, Gabriela Echeverría-Garcés, Luis Sullón-Dextre, Eugenia Cordero-García, Alexis R. Morales, Andrea Avendaño, Enrique Sánchez, Laura C. Bastone, Cesar Lara, Patricia Zuluaga-Arias, Ana María Soler, Julio Da Luz, Gabriela Burgueño-Rodríguez, Marcelo Vital, Elizabeth Reyes-Reyes, Alexander Huaccha, Yeimy V. Ariza, Naomi Tzul, Ana L. Rendón, Roberto Serrano, Larissa Acosta, Angelo Motta-Pardo, Leonardo Beltrán-Angarita, Erika Brand, Miguel A. Jiménez, Gladys Maribel Hidalgo-Lozada, Marina M. J. Romero-Prado, Karla Escobar-Castro, Mariel Umaña-Rivas, Juan D. Vivas, Paola Lagos, Yineth Ballén Martínez, Sharleth Quesada, Camila Calfio, Maria L. Arias, María A. Lavanderos, Dante D. Cáceres, Alberto Salazar-Granara, Nelson M. Varela, Luis A. Quiñones

**Affiliations:** ^1^ Department of Pharmacology, Toxicology and Pharmaco-Dependence, Faculty of Pharmacy, University of Costa Rica, San Jose, Costa Rica; ^2^ Laboratory of Chemical Carcinogenesis and Pharmacogenetics, Department of Basic-Clinical Oncology (DOBC), Faculty of Medicine, University of Chile, Santiago, Chile; ^3^ Cancer Research Group (CRG), Faculty of Medicine, Universidad de Las Américas, Quito, Ecuador; ^4^ Molecular Diagnostic Laboratory, Genetics Division, Faculty of Medicine, Hospital de Clínicas José de San Martín, University of Buenos Aires, Buenos Aires, Argentina; ^5^ Universidad del Rosario, School of Medicine and Health Sciences, Center for Research in Genetics and Genomics (CIGGUR), Institute of Translational Medicine (IMT), Bogotá, Colombia; ^6^ Molecular Genetic Unit, School of Chemistry, Universidad de la República, General Flores, CP 1800 2124, Montevideo, Uruguay; ^7^ Toxicology and Public Health Research Laboratory, Department of Research, Faculty of Dentistry, University of Cartagena, Cartagena, Colombia; ^8^ Academy of Genomics and Laboratory of Pharmacogenomics and Molecular Biomedicine, Instituto Politécnico Nacional, CIIDIR-Unidad Durango, Durango, Mexico; ^9^ Instituto de Fisiopatología y Bioquímica Clínica (INFIBIOC), Facultad de Farmacia y Bioquímica, Universidad de Buenos Aires- Argentina, Buenos Aires, Argentina; ^10^ National Centre for Quality Control of Drugs, La Havana, Cuba; ^11^ Department of Pharmaceutical Sciences and Technology, Faculty of Chemical and Pharmaceutical Sciences, University of Chile, Santiago, Chile; ^12^ Department of Molecular Biology, Galileo University, Guatemala City, Guatemala; ^13^ Department of Health. Faculty of Medicine, Federal University of Mato Grosso (UFMT), Cuibá, Brazil; ^14^ Morphology and Applied Immunology Research Center (NuPMIA), University of Brasilia (UnB), Brasília, Brazil; ^15^ Department of Microbiology, Faculty of Biochemical and Pharmaceutical Sciences. National University of Rosario, Rosario, Argentina; ^16^ Laboratory Pathological Anatomy, Hospital Luis Calvo Mackenna, Santiago, Chile; ^17^ Department of Research in Pharmacogenomics, Faculty of Chemical Sciences and Pharmacy, University of San Carlos de Guatemala, Guatemala, Guatemala; ^18^ Clinical Laboratory Blood Biochemistry and Immunoassay Section, Hospital Clínico Félix Bulnes Cerda, Santiago, Chile; ^19^ Pharmacy Service, Hospital UC-Christus, Santiago, Chile; ^20^ Medical Genetics Platform, Santa Fe, Argentina; ^21^ Facultad de Ciencias de La Salud, Universidad Internacional de Valencia, Valencia, España; ^22^ Sociedad de Farmacología Molecular del Perú, Lima, Perú; ^23^ Department of Toxicology and Pharmacology, Faculty of Pharmacy and Bioanalisis, University of Los Andes, Merida, Venezuela; ^24^ Department of Pediatrics, Medical Genetics Unity, Faculty of Medicine, University of Los Andes, Mérida, Venezuela; ^25^ Vivian Pellas Hospital, Managua, Nicaragua; ^26^ Laboratorio de Medicina Genómica, Gammalab, Grupo Gamma, Rosario, Argentina; ^27^ Betesda La Alternativa Natural, San José, Costa Rica; ^28^ Colombian Association of Pharmacovigilance, Bogotá, Colombia; ^29^ Laboratorio de Genética Molecular Humana, Departamento de Ciencias Biológicas, Universidad de La República, Montevideo, Uruguay; ^30^ Molecular Genetic Unit, School of Chemistry, Universidad de la República, General Flores 2124, Montevideo, Uruguay; ^31^ Clinical Experimental Pharmacology Section, Teaching and Research Department, Institute of Oncology and Radiobiology, Havana, Cuba; ^32^ Pharmacy Service, Hospital II 1 Moyobamba, San Martín, Perú; ^33^ Pharmaceutical Chemistry Program, El Bosque University, Bogotá, Colombia; ^34^ Drug Inspectorate Unit, Ministry of Health and Wellness, Belmopan, Belize; ^35^ Department of Pharmaceutical Technology, National Autonomous University of Honduras, Tegucigalpa, Honduras; ^36^ PhV Latam, San Salvador, El Salvador; ^37^ Faculty of Biology, Chemistry and Pharmacy, Galileo University, Guatemala, Guatemala; ^38^ GENOBIDC, Faculty of Pharmacy and Biochemistry, Universidad Nacional Mayor de San Marcos (UNMSM), CIGBM, Faculty of Medicine, Universidad de San Martin de Porres (USMP), Lima, Peru; ^39^ Faculty of Heath Sciences, Central Unit of Valle del Cauca, Tuluá, Colombia; ^40^ Chemistry School, Universidad Tecnológica de Pereira, Pereira, Colombia; ^41^ Postgraduate Department, Master’s Degree in Immunology, Universidad Cayetano Heredia, Lima, Perú; ^42^ Clinical Pharmaceutical Services, Health Division, Chillán, Chile; ^43^ Department of Physiology, Health Sciences University Center, University of Guadalajara, Guadalajara, Mexico; ^44^ Department of Physiology, Health Sciences University Center, University of Guadalajara, Guadalajara, Jalisco, Mexico; ^45^ Laboratory of Histocompatibility and Immunogenetics, Department of Nephrology and Transplant, Hospital General San Juan de Dios, Guatemala, Guatemala; ^46^ Department of Tropical Medicine, Faculty of Medicine, University of Brasília, Brasília, Brazil; ^47^ Department of Clinical Pharmacology, Faculty of Medicine, University of La Sabana, Chía, Colombia; ^48^ Recombinant Biopharmaceutical Laboratory, Department of Pharmacology, Faculty of Biological Sciences, University of Concepción, Concepción, Chile; ^49^ Medical Specialist in Epidemiology Clinical Pharmacology Teacher; Medical Scientific and Pharmacovigilance Advisor in the Pharmaceutical Industry, Bogotá, Colombia; ^50^ Faculty of Pharmacy, University of Costa Rica, San Jose, Costa Rica; ^51^ International Center for Biomedicine ICC, Santiago, Chile; ^52^ Tropical Diseases Research Center and Microbiology Faculty, University of Costa Rica, San José, Costa Rica; ^53^ Environmental Health Programme, School of Public Health, Faculty of Medicine, University of Chile, Santiago, Chile; ^54^ Universidad de San Martín de Porres, Facultad de Medicina Humana, Centro de Investigación de Medicina Tradicional y Farmacología, Lima, Perú

**Keywords:** pharmacogenetics, pharmacogenomics, gene/drug pair, barriers, precision medicine

## Abstract

Pharmacogenomics (PGx) is considered an emergent field in developing countries. Research on PGx in the Latin American and the Caribbean (LAC) region remains scarce, with limited information in some populations. Thus, extrapolations are complicated, especially in mixed populations. In this paper, we reviewed and analyzed pharmacogenomic knowledge among the LAC scientific and clinical community and examined barriers to clinical application. We performed a search for publications and clinical trials in the field worldwide and evaluated the contribution of LAC. Next, we conducted a regional structured survey that evaluated a list of 14 potential barriers to the clinical implementation of biomarkers based on their importance. In addition, a paired list of 54 genes/drugs was analyzed to determine an association between biomarkers and response to genomic medicine. This survey was compared to a previous survey performed in 2014 to assess progress in the region. The search results indicated that Latin American and Caribbean countries have contributed 3.44% of the total publications and 2.45% of the PGx-related clinical trials worldwide thus far. A total of 106 professionals from 17 countries answered the survey. Six major groups of barriers were identified. Despite the region’s continuous efforts in the last decade, the primary barrier to PGx implementation in LAC remains the same, the “need for guidelines, processes, and protocols for the clinical application of pharmacogenetics/pharmacogenomics”. Cost-effectiveness issues are considered critical factors in the region. Items related to the reluctance of clinicians are currently less relevant. Based on the survey results, the highest ranked (96%–99%) gene/drug pairs perceived as important were *CYP2D6*/tamoxifen, *CYP3A5*/tacrolimus, *CYP2D6*/opioids, *DPYD*/fluoropyrimidines, *TMPT*/thiopurines, *CYP2D6*/tricyclic antidepressants, *CYP2C19*/tricyclic antidepressants, *NUDT15*/thiopurines, *CYP2B6*/efavirenz, and *CYP2C19*/clopidogrel. In conclusion, although the global contribution of LAC countries remains low in the PGx field, a relevant improvement has been observed in the region. The perception of the usefulness of PGx tests in biomedical community has drastically changed, raising awareness among physicians, which suggests a promising future in the clinical applications of PGx in LAC.

## 1 Introduction

As a highly actionable form of precision medicine, pharmacogenomics (PGx), a discipline that studies the impact of germline and somatic genetic variations on drug response, is constantly evolving. Several studies have estimated that more than 80% of the global population carries at least one actionable pharmacogenomic (PGx) variant, *i.e.*, a genetic variation that confers increased risk of toxicity and/or decreased efficacy when treated with a particular drug that could be prevented or mitigated if the risk were known (McInnes et al., 2014; Bush et al., 2016; Mostafa et al., 2019; Van Driest et al., 2021; ClinGen, 2023). This affects prescribing decisions to change the drug or dose, facilitating the transition to patient-specific drug regimens and thus improving efficacy and reducing toxicity (Van Driest et al., 2014; Bush et al., 2016; Mostafa et al., 2019; McInnes et al., 2021). This field is considered emergent in developing countries. In this respect, research on PGx in the Latin American region is growing but remains scarce, with limited information in some populations. Thus, extrapolations of PGx recommendations are complicated, especially in mixed populations (Abou et al., 2019; Ayati et al., 2021; Medwid and Kim, 2022).

A number of studies worldwide have demonstrated the cost-effectiveness of PGx testing, supporting its clinical application, particularly in public institutions (Stallings et al., 2006; Verbelen et al., 2017; Wang et al., 2018; Plumpton et al., 2019; Zhu et al., 2020 and; Zhu, 2021; Sukri et al., 2022). In this respect, major PGx expert organizations such as the Clinical Pharmacogenetics Implementation Consortium (CPIC) (Relling et al., 2020) and the Dutch Pharmacogenetics Working Group (DPWG) (Swen et al., 2008) provide guidelines for PGx clinical implementation of “actionable variants”. Indeed, the Food and Drug Administration (FDA) has published a list of PGx biomarkers for drug labeling with pharmacogenetically guided dosing (FDA, 2022).

The application of PGx in the Latin American population presents several challenges, including the heterogeneity of the genetic admixtures, the socioeconomic context of each country and the differences in the health strategies implemented, among others. Arguably, the Latin American population is the most recently mixed and heterogeneous population worldwide, which reflects a history of massive settlement by immigrants (primarily Spaniards) and their variable admixture. In addition, the diversity of Amerindians since the Columbus voyages in 1492 and then the slave trade from Africa contribute to the heterogeneity. Today, Latin America has variable admixtures of African, European and Native American populations. The genetic variation of this region is underrepresented in genetic databases worldwide, and evidence related to PGx for these ethnicities is currently insufficient. This context is particularly relevant in PGx studies, where the phenotypic response to drugs is sometimes significantly different from that observed in homogeneous populations. Thus, Latin America can be seen as an ideal population for PGx studies in which polymorphic loci and linkage disequilibrium can be used to infer the genetic basis of drug response (Eyheramendy et al., 2015; Homburger et al., 2015; Adhikari et al., 2016; López-Cortés et al., 2017; Chacón-Duque et al., 2018; Norris et al., 2018; Verdugo et al., 2020; Varela et al., 2021; De Oliveira et al., 2023; Statista, 2023).

Despite several individual and collective efforts in LAC to implement PGx in clinical practice, the transition represents a relevant challenge due to genetic/pharmacogenetic diversity, political variables and idiosyncratic perspectives. The perceived importance of barriers to implement PGx testing in clinical practice, examined in 2014 for LAC, as all over the world, seems to be still very relevant, i.e., a) the need of clear guidelines for the use of PGx in clinical practice, b) the insufficient awareness of PGx among clinicians, and c) the absence of regulatory institutions that enables the implementation of pharmacogenetic tests (Quiñones et al., 2014).

In 2014, a group of Latin American researchers was established to address the problem (Quiñones et al., 2014), and in 2015, the group was consolidated into the “First Latin American Congress of Pharmacogenomics and Personalized Medicine” in Viña del Mar, Chile (May 21 to 23, 2015), forming the Latin American Society of Pharmacogenomics and Personalized Medicine (SOLFAGEM) (www.solfagem.org). This society aims to strengthen the development of PGx scientific research, both theoretical and experimental, to increase the awareness and promote the dissemination of the discipline through knowledge creation, clinical tool searches, and the identification of products or biomarkers that can improve current disease treatments, as well as any other initiative conducive to a broad utilization of this scientific discipline for the benefit of Latin America and the Caribbean (LAC) and global public health. In 2015, the first president of SOLFAGEM became a member of CPIC to represent LAC countries and learn about the CPIC guidelines and procedures. However, no review of CPIC guidelines or contributions has been requested from any of SOLFAGEM’s presidents up to this point. Afterwards, in March 2019, the Ibero-American Program on Science and Technology for Development (CYTED) network called RELIVAF (Latin American Network for Validation and Implementation of Pharmacogenomic Clinical Guidelines), with the participation of 12 countries, was born. The primary goal of this organization is to create a network of Latin American basic-clinical research centers in PGx to establish an exchange and to generate discussion groups about the configuration of specific (customized) clinical pharmacogenomics implementation Guidelines for LAC, according to ethnic variability, to be used in regional health systems.

In this respect, given that the majority of the authors first examining PGx testing and the barriers to clinical application in 2014 are now members of RELIVAF, we present here an updated assessment of LAC after 8 years of joint efforts to improve the PGx knowledge and its clinical applications. In addition, a renewed exploration of the contribution of Latin America to global PGx knowledge is included.

## 2 Methods

**FIGURE 1:**
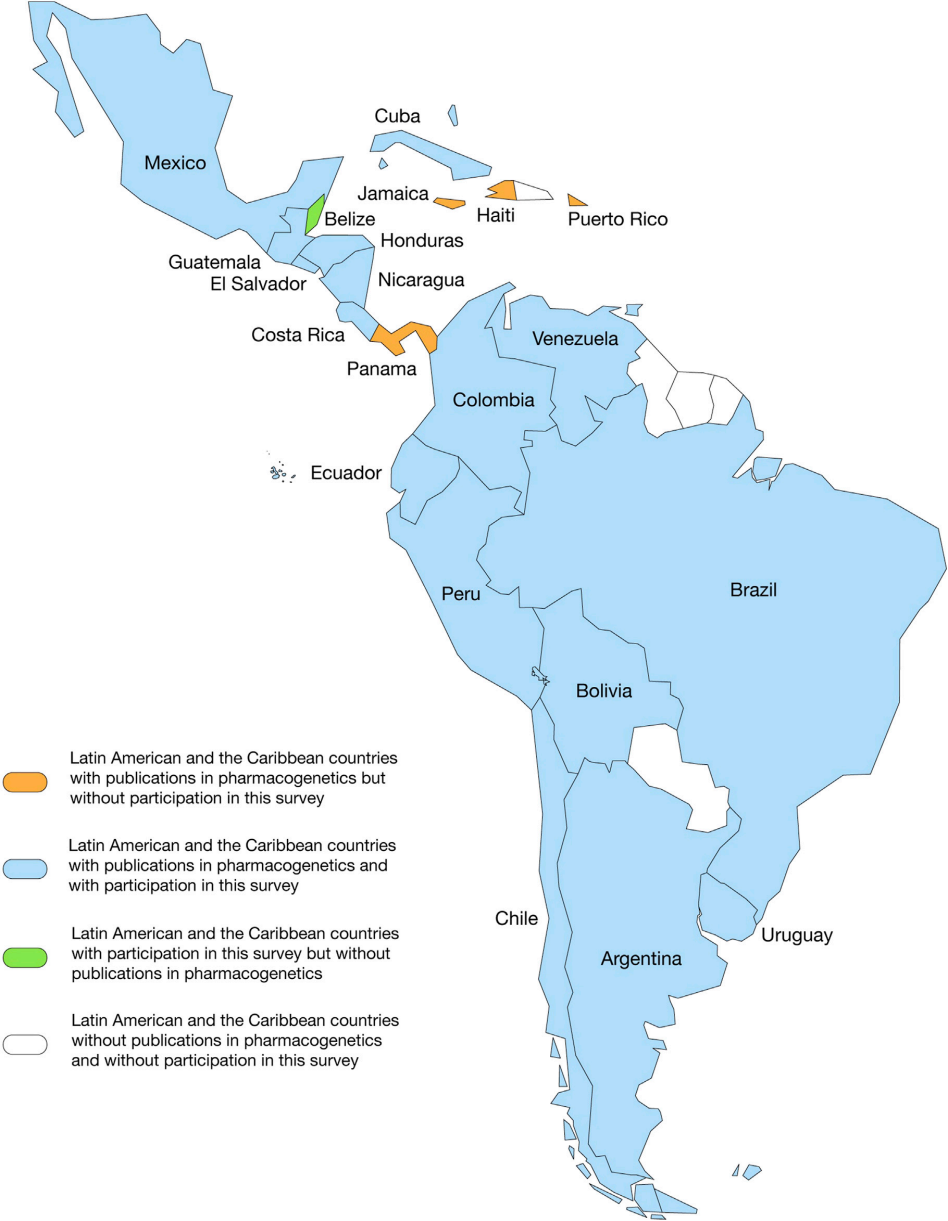
Panoramic landscape of Latin American and the Caribbean countries with publications in the pharmacogenomics field and with participation in this survey.

**FIGURE 2:**
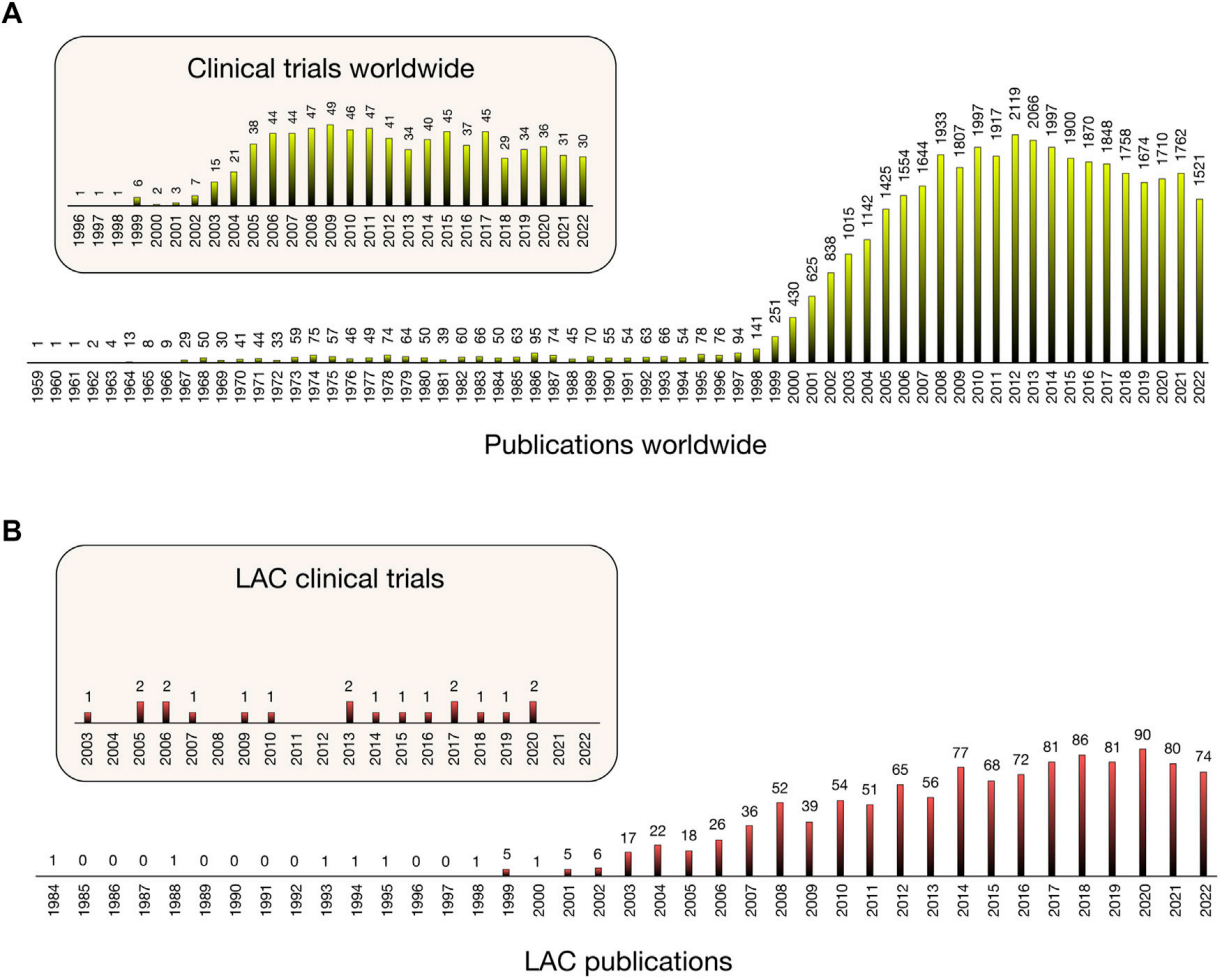
Variation in number of publications [Scopus] and clinical trials [Clinicaltrials.gov] including pharmacogenomics/pharmacogenetics studies from 1959 worldwide **(A)** and from 1984 in Latin America and the Caribbean **(B)**.

To understand the evolution of the PGx field in LAC and the contribution of LAC countries to global PGx advances, a search was conducted for publications from 1 January 1959, to 1 November 2022, using the Scopus database and other complementary sources (PubMed, Cochrane, Lilacs, Scielo) with the following PGx-related keywords: polymorphisms, pharmacogenetics, pharmacogenomics, SNPs, biomarkers, adverse drug reactions, precision medicine, personalized medicine, and clinical pharmacology. The keywords were used in a logical combinatorial manner to ensure they adjusted to the field. First, we (AS-H, LAQ, MG and MC) analyzed abstracts and then, when necessary, the full text of manuscripts, to ensure their cover topics within the field. This approach yielded 38,786 publications worldwide and 1,337 from 20 LAC countries (starting in 1984). Publications from Antigua and Barbuda, Dominican Republic, Guyana, Paraguay, Suriname, and Trinidad and Tobago were not found (Figure 1; Figure 2). Completed clinical trials applying the PGx concept, that is, screening and enrollment by genotype or analysis of the results in light of patient genotype, were also included in this work. This search was performed on the clinicaltrials. gov web page.

Using the results of the publication’s search and complementing this with members of LAC scientific associations and societies (RELAGH-“Red Latino Americana de Genética Humana”-; ALF-“Asociación Latino Americana de Farmacología”-; ALAG-“Asociación Latinoamericana de genética”- and RELIVAF) and the authors of this manuscript, we identified 188 potential respondents, who were contacted to answer a structured survey (Supplementary Material) in order to evaluate the perception of the usefulness of drug/gene pairs and barriers to pharmacogenomics in Latin America and the Caribbean. A total of 106 biomedical professionals from 17 countries answered the survey (response rate: 56.4%) (Table 1). Survey questions were adapted from the initially published manuscript in 2014 (Quiñones et al., 2014) to properly compare the results of both surveys in order to evaluate advances in the field. The adaptation, besides minor writing changes, consisted of the following: we used the same 16 potential barriers as the 2014 survey, which were replicated originally from CPIC and Spain surveys (Relling and Klein, 2011; Agúndez et al, 2012), we added one additional potential barrier that we considered especially relevant for LAC (Lack of cost-effectiveness of pharmacogenomic studies) and we excluded three original barriers, that is, “Insufficient development of processes and protocols for PGx use” and “Insufficient definition of the clinical impact of SNPs on specific drugs” because these were considered included in others barriers and/or were considered not relevant enough today, according to the general consensus in the field. Similarly, we excluded “Insufficient PGx characterization of the target population” because we considered this to be included in “Insufficient characterization of pharmacogenomic variability in Latin American populations”, thus, we intended to avoid overlapping questions.

**TABLE 1:** General characteristics of the survey respondents (n = 106).

	n (%)
Sex	
Female	65 (61.3)
Male	41 (38.7)
Countries	
Chile	19 (17.9)
Costa Rica	16 (15.1)
Guatemala	14 (13.2)
Argentina	11 (10.4)
Colombia	10 (9.4)
Mexico	8 (7.5)
Uruguay	6 (5.7)
Peru	5 (4.7)
Cuba	4 (3.8)
Ecuador	3 (2.8)
Brazil	2 (1.9)
El Salvador	2 (1.9)
Honduras	2 (1.9)
Belize	1 (0.9)
Bolivia	1 (0.9)
Nicaragua	1 (0.9)
Venezuela	1 (0.9)
Professional profiles	
Pharmacist	46 (43.4)
Biochemist	18 (17.0)
Medical Doctors	14 (13.2)
Biologist	10 (9.4)
Biotechnologist	5 (4.7)
Medical technologist	3 (2.8)
Nutritionist	3 (2.8)
Toxicologist	2 (1.9)
Microbiologist	2 (1.9)
Chemist	2 (1.9)
Bioinformatician	1 (0.9)
Institutions	
Universities	58 (54.7)
Clinical Centres	38 (35.8)
Research Centres	6 (5.7)
Sanitary Authorities	2 (1.9)
Pharmaceutical Industry	2 (1.9)

**FIGURE 3:**
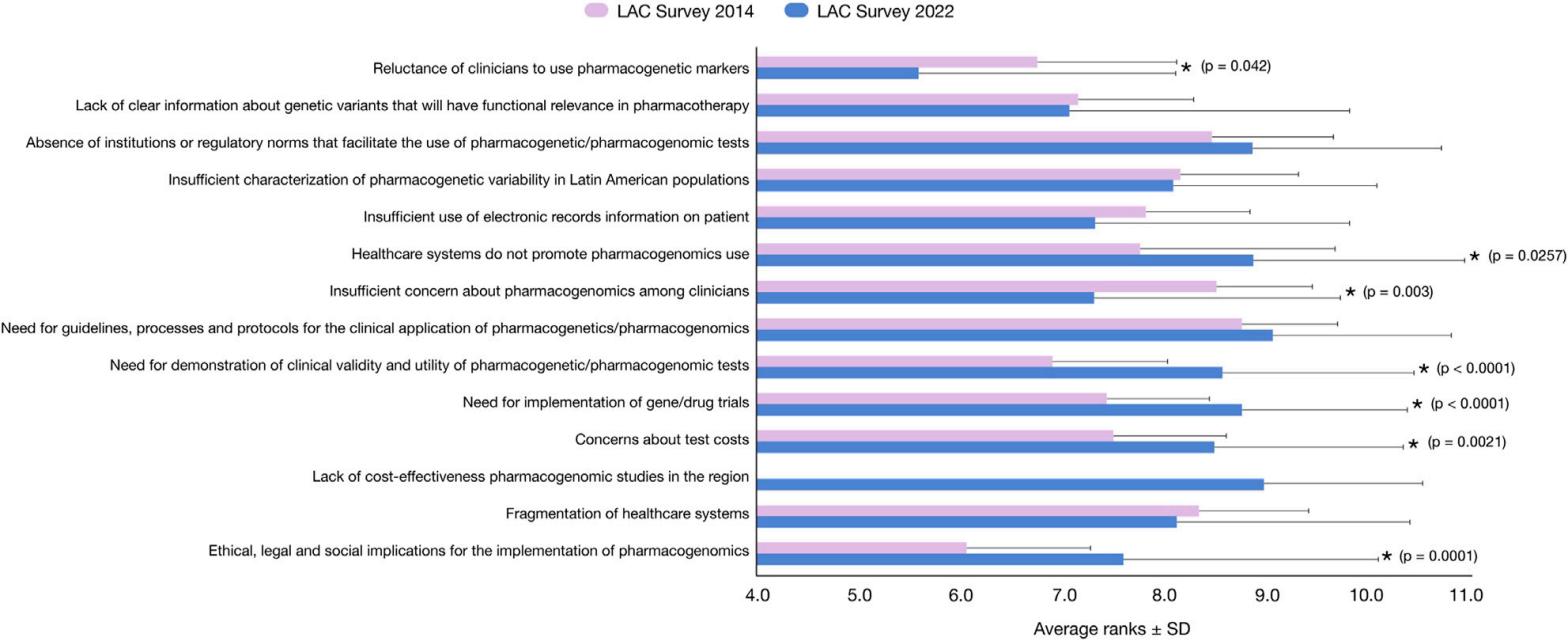
Highest-ranking barriers for implementing the use of pharmacogenomics testing, based on a survey in Latin American and the Caribbean scientific and clinical Researchers in 2022 (in blue) compared to the 2014 survey (in pink) (Quiñones et al., 2014 **)**. Data related to average importance on a scale of 1 (low) to 10 (high) ± standard deviation are plotted along the X-axis. *p* < 0.05 (unequal variance *t*-test) is statistically significant (indicated with a star).

**FIGURE 4:**
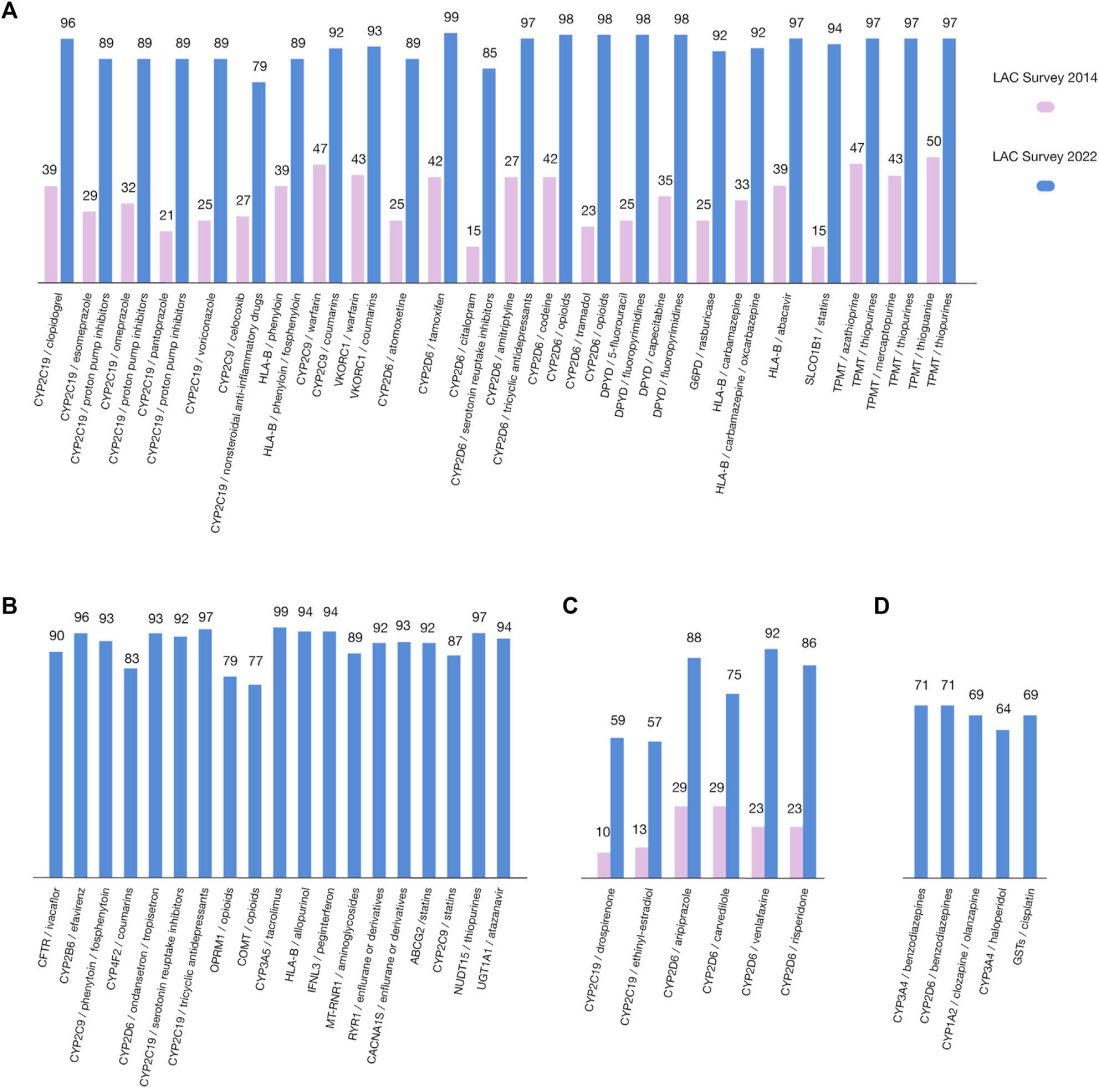
Highest-ranking gene/drug pairs, based on the survey of the Latin American scientific and clinical community (blue), compared to the previous published survey in 2014 (pink). Data related to the percentages of respondents who ranked the gene/drug pairs as 3, 4, and 5 in relation to the total evaluations (on a scale of 1(low)–5(high)) are plotted along the Y-axis. **(A)** 2022/2014 comparison of CPIC gene/drug pairs; **(B)** CPIC gene/drug pairs not included in 2014 survey; **(C)** 2022/2014 comparison of non-CPIC gene/drug pairs; **(D)** Non-CPIC gene/drug pairs not included in 2014 survey.

Like in 2014, the final survey was structured into two segments. First, a list of 14 potential barriers to PGx biomarkers’ clinical application was evaluated with respect to their importance on a scale from 1 to 10 (10 being the highest) and the average ± SD of all respondents for each barrier is reported (Figure 3). Second, a list of 54 gene/drug pairs was evaluated on a scale of 1–5 (5 being the highest) to evaluate an association between biomarkers and their response to genomic precision medicine (Figure 4). This scale and its evaluation are the same used before by CPIC, Spain and LAC surveys, for comparison purposes (Relling and Klein, 2011; Agúndez et al., 2012; Quiñones et al., 2014) Of the 54 total gene/drug pairs, we evaluated 43 CPIC pairs (CPIC, 2022) and 11 non-CPIC pairs, which could be relevant to LAC populations according to RELIVAF-members opinion. It should be noted that not all included CPIC pairs actually have specific actionable guidelines (e.g., OPRMI-opioids and COMT-opioids). The comparison between the importance of barriers from the 2014 and 2022 surveys was performed through the two-sample *t*-test with unequal variance (STATA 17.0) using number of responses, average and standard deviation of each survey and *p* < 0.05 as statistically significant (Table 2).

**TABLE 2:** Comparison of the importance of barriers between the 2014 and 2022 surveys in LAC.

Survey question	2014 (average ± S.D.) n = 20	2022 (average ± S.D.) n = 106	p-value*
Reluctance of clinicians to use pharmacogenetic markers	6.748 ± 1.356	5.575 ± 2.522	**0.0042**
Lack of clear information about genetic variants that will have functional relevance in pharmacotherapy	7.141 ± 1.117	7.057 ± 2.732	0.8173
Absence of institutions or regulatory norms that facilitate the use of pharmacogenetic/pharmacogenomic tests	8.454 ± 1.184	8.849 ± 1.845	0.2240
Insufficient characterization of pharmacogenetic variability in Latin American populations	8.141 ± 1.153	8.075 ± 1.994	0.8397
Insufficient use of electronic records information on patient	7.798 ± 1.031	7.311 ± 2.486	0.1497
Healthcare systems do not promote pharmacogenomics use	7.748 ± 1.908	8.858 ± 2.063	**0.0257**
Insufficient concern about pharmacogenomics among clinicians	8.497 ± 0.933	7.302 ± 2.403	**0.0003**
Need for clear guidelines, processes and protocols for the clinical application of pharmacogenetics/pharmacogenomics in LAC	8.748 ± 0.914	9.057 ± 1.739	0.2508
Need for demonstration of clinical validity and utility of pharmacogenetic/pharmacogenomic tests	6.890 ± 1.123	8.557 ± 1.867	**< 0.0001**
Need for implementation of gene/drug trials	7.423 ± 0.994	8.745 ± 1.622	**< 0.0001**
Concerns about test costs	7.485 ± 1.104	8.481 ± 1.837	**0.0021**
Lack of cost-effectiveness pharmacogenomics studies in the region	N.D.	8.962 ± 1.549	N.A
Fragmentation of healthcare systems	8.325 ± 1.074	8.104 ± 2.284	0.5010
Ethical, legal and social implications for the implementation of pharmacogenomics	6.049 ± 1.215	7.585 ± 2.491	**0.0001**

*Two-sample student test with unequal variances. N.D: No data (not requested in 2014), N.A: not applicable, S.D: standard deviation, *p* < 0.05 is considered as significant (in bold).

## 3 Results

### 3.1 Determination of LAC countries’ contributions to PGx research

Our search for PGx publications revealed that the field is a hotspot research area, giving rise to almost 39,000 publications since Dr. Friedrich Vogel’s first publication, where the term pharmacogenetics was coined and defined (Vogel, 1959). Twenty LAC countries (Figure 1) have contributed 3.44% (1,337) to these publications since 1984 (Figure 2A). More significant LAC contributions were from Brazil (41.96%), México (17.28%), Chile (8.98%) (7.78%) and Colombia (6.81%) (Supplementary Material). Similarly, of the total 774 completed PGx-related clinical trials worldwide from 1996, 19 were from the LAC region from 2003 (2.45%) (Figure 2B). Clinical trials have been performed with the participation of Brazil, Chile, Argentina, México, Perú, Uruguay and Puerto Rico.

### 3.2 Assessing perceptions and barriers of PGx in LAC countries

Figures 3, 4 summarize the results of the LAC survey of the perceived importance of barriers to the implementation of PGx testing in clinical practice (on a scale from 1 to 10). Of the total respondents, the distribution was as follows: 17.9% from Chile, 15.1% from Costa Rica, 13.2% from Guatemala, 10.4% from Argentina, 9.4% from Colombia, 7.5% from México, 5.7% from Uruguay, 4.7% from Perú, 3.8% from Cuba, 2.8% from Ecuador, 1.9% each from Brazil, El Salvador and Honduras, and 0.9% each from Belize, Bolivia, Nicaragua and Venezuela. Of these respondents, 61.3% were women. We could not obtain responses from Puerto Rico, Panamá, Jamaica or Haití. The professional profile of the participants was as follows: 43.4% pharmacists, 17.0% biochemists, 13.2% medical doctors, 9.4% biologists, 4.7% biotechnologists, 2.8% medical technologists, 2.8% nutritionists, 1.9% toxicologists, 1.9% microbiologists, 1.9% chemists and 0.9% bioinformaticians. Participant affiliations varied between universities (54.7%), clinical centers (35.8%), research centers (5.7%), sanitary authorities (1.9%) and the pharmaceutical industry (1.9%) (Table 1).

The results observed in Figure 3 revealed six major groups of barriers: 1) “need for guidelines, processes and protocols for the clinical application of pharmacogenetics/pharmacogenomics” (9.06 points), which is not significantly different from the previously reported score in for similar barrier evaluated in 2014 (8.76 points); 2) “lack of cost-effectiveness pharmacogenomics studies in the region” (8.96 points), an item not included in the 2014 survey; 3) “healthcare systems do not promote pharmacogenomics use” (8.86 points), which is slightly higher than in the 2014 survey (7.69 points); 4) “absence of institutions or regulatory norms that facilitate the use of pharmacogenetics/pharmacogenomic tests” (8.85 points), not significantly different from the 2014 survey (8.45 points); 5) “need for implementation of gene/drug trials” (8.74 points), which is significantly higher than previously reported (7.43 points) (*p* < 0.001); and 6) “need for demonstration of clinical validity and utility of pharmacogenetic/pharmacogenomic tests” (8.55 points), which is significantly higher than the 2014 survey (6.92 points) (*p* < 0.001). Additionally, the “Reluctance of clinicians to use pharmacogenetic markers” and the “insufficient concern about pharmacogenomics among clinicians” were significantly lower in the present survey compared with 2014 (5.78 versus 6.75 points; *p* = 0.042 and 8.50 v*ersus* 7.30 points; *p* = 0.003, respectively). Conversely, the “Ethical, legal and social implications of pharmacogenomics” and the “Concerns about the test costs” were significantly higher than in 2014 (7.58 *versus* 6.05 points; *p* = 0.001 and 7.48 *versus* 8.48 points; *p* = 0.021, respectively).

As previously mentioned, in the present survey, we also included 54 gene/drug pairs for evaluation (43 CPIC pairs and 11 non-CPIC pairs). These pairs include the same 51 pairs evaluated in 2014. However, some are now grouped by CPIC (e.g., mercaptopurine, azathioprine, and thioguanine, now grouped as “thiopurines”). Thus, the number of CPIC pairs does not perfectly match between the surveys (Figure 4), the highest ranked CPIC pairs according to the number of respondents with respect to the perceived importance of the data linking the drug to the gene variation were *CYP2D6*/tamoxifen (99), *CYP3A5*/tacrolimus (99), *CYP2D6*/opioids (98), *DPYD*/fluoropyrimidines (98) and *TMPT*/thiopurines (97), *CYP2D6*/tricyclic antidepressants (97), *CYP2C19*/tricyclic antidepressants (97), *NUDT15*/thiopurines (97), CYP2B6/efavirenz (96), and CYP2C19/clopidogrel (96) (Figures 4A, B). With respect to the evaluated non-CPIC pairs, the highest-ranked pairs were CYP2D6/venlafaxine (92), CYP2D6/aripiprazole (88), and CYP2D6/risperidone (85) (Figures 4C, D).

## 4 Discussion

The PGx field has been growing since the first contributions of Drs. Friedrich Vogel and Arno Motulski (Vogel, 1959 and; Vogel, 1961; Motulski, 1960). However, LAC contributions remain scarce with respect to publications (3.44%), application in clinical trials (2.45%), and clinical practice tools. Furthermore, a worldwide total of twenty-three journals in the PGx field (NCBI, NML Catalog, 2023) and ninety-three with at least one related section (PubsHub, 2023) already exist, none of which are from Latin America. However, at least two books (Quiñones et al., 2017; Quiñones (ed.) and Redal (ed.), 2020) and several chapters about PGx in Latin America or from LAC researchers have been published in recent years (Ríos-Santos, 2012; Martínez & Quiñones, 2018; Cayún & Quiñones, 2019; Cerpa et al., 2021; Varela et al., 2021; Martínez et al., 2021a; Martínez et al. b; Quiñones et al., 2021). Moreover, some recently formed regional scientific efforts, for example, the Latin American Society for Pharmacogenomics and Personalized Medicine (SOLFAGEM, in 2015), the Latin American Network for Implementation and Validation of Clinical Pharmacogenomics Guidelines (RELIVAF- CYTED, in 2019), and the Latin American Network of Human Genetics (RELAGH, in 2014), are working to minimize the evidence gap and increase PGx information. However, the implementation of PGx in clinical practice remains a significant challenge in LAC due to the heterogeneity of countries with respect to socioeconomic level and political, sanitary, and administrative strategies. To date, LAC countries do not have biorepositories, biobanks and/or databases to provide samples and data for PGx purposes, which is a critical need in the region (Vargas and Cobar, 2021). In addition, the publication of any PGx specific clinical guideline has not been performed or adapted for LAC region. Nonetheless, as a middle-income region, with the availability of basic molecular tools in most of the countries of the region, PGx implementation is possible. To promote implementation, RELIVAF, an initiative to which all the authors of this manuscript belong to or collaborate with, pursues the following objectives: 1) to collect scientific information, strategies, and new perspectives, especially in countries with poor PGx development; 2) to generate dissemination/education activities in the region; 3) to potentiate collaborative research among countries; and 4) to set up feasible clinical implementation tools for Latin American patients (Quiñones & Redal, 2020; Esperón et al., 2022). New research continues with the integration and implementation of pharmacogenomics in the region. Furthermore, progress is due to advances in technologies and the combination of different specialties through interdisciplinary work.

Since 2014, some educational advances in LAC could be mentioned, as, for example, at least 9 local undergraduate and postgraduate courses in Argentina, Brazil, Costa Rica, Chile, Guatemala, Mexico, Perú and Uruguay, most of them with participation of RELIVAF-members (see supplementary material). One international RELIVAF-Latin American postgraduate program recently finished (https://relivaf.mailchimpsites.com). Besides, the setting-up of two specific laboratories in Guatemala and Colombia by RELIVAF members (Drs. Rodrigo Vargas and Farith Gonzalez, respectively), a Guideline for PGx studies in Cuba (CECMED, 2023) and the incorporation of PGx testing in several laboratories and clinical centers of the region (Biolinks, 2023; Biopharm, 2023; CLC, 2023; GM, 2023; UFF, 2023). Moreover, the Brazilian biggest diagnostic company DASA Genomica has recently launched PharmOneTM a pharmacogenomic tool for personalized medicine (DASA Genomica, 2023). Finally, SOLFAGEM has organized 5 Congress in different countries (Viña del Mar-Chile, 2015; Durango-Mexico, 2017; Cusco-Perú, 2019; Buenos Aires-Argentina, 2021 and Cartagena-Colombia, 2023) to support the development in the field.

Therefore, in this paper, we examined the advance, in almost a decade, in the knowledge concerning pharmacogenomic tests and the barriers to their clinical application among members of the LAC scientific and clinical community. We conducted a simple and structured regional survey that evaluated a list of possible barriers to the clinical implementation of biomarkers based on their importance, as performed in the past by CPIC, Spain an LAC researchers. In addition, a paired list of genes/drugs was analyzed to determine an association between biomarkers and response to genomic medicine. This survey was compared to our previous survey (Quiñones et al., 2014) to assess progress in the region, to understand the present situation and to address efforts to improve clinical implementation in LAC.

The information collected from 106 respondents from 17 countries means a significant increase in comparison to the 2014 survey, wherein the information was obtained from only 20 respondents from 13 countries. Therefore, the number of publications from LAC in the field and properly informed professionals have increased considerably in the last 8 years.

Despite the region’s continuous efforts in the last decade, the primary barrier to PGx implementation in LAC remains the same, *i.e.*, the “need for guidelines, processes, and protocols for the clinical application of pharmacogenetics/pharmacogenomics” for the region (Figure 3). Some items are considered more relevant today than in 2014; for example, “Healthcare systems do not promote pharmacogenomics use” (7.69 vs. 8.86 points), “Absence of institutions or regulatory norms that facilitate the use of pharmacogenetics/pharmacogenomic tests” (8.45 vs. 8.85 points), and “need for implementation of gene/drug trials” (7.43 vs. 8.74 points). Similarly, the “need for demonstration of clinical validity and utility of pharmacogenetic/pharmacogenomic tests” increased significantly, from 6.92 points to 8.55 points. Together, these results suggest that guidelines, protocols, norms, and clinical trials associated with PGx are considered critical milestones of the implementation of PGx in LAC healthcare systems. Concomitantly, it appears that biomedical professionals are considerably more aware of the field.

Interestingly, the new item “Lack of cost-effectiveness pharmacogenomics studies in the region” not included in the 2014 survey was considered the second-most relevant barrier (8.96 points) in the present survey. This indicator reflects significant concern about the feasibility of implementing PGx in LAC, which agrees with the results of the item “concern about test costs,” which increased by 1.00 point from the 2014 to the 2022 survey (7.48–8.48). In developing countries, the cost factor is a persistent obstacle in the implementation of new technologies. It is generally believed that if it is possible to demonstrate the cost effectiveness of PGx implementation in daily clinical practice, the effect of other obstacles derived from cost effectiveness would be mitigated, such as the lack of its promotion in health systems.

However, significant advances should be noted; for example, the “reluctance of clinicians to use pharmacogenetic markers” is now less relevant, decreasing significantly from 6.7 points in 2014 to 5.6 in 2022. This change may be related to the knowledge and awareness of the field, which is supported by both the significant decrease in the “insufficient concern about PGx among clinicians” from 8.5 points in 2014 to 7.3 points in 2022 and the significant increase in the concern about the “ethical, legal and social implications for the implementation of PGx” from 6.0 points in 2014 to 7.6 points in 2022. In addition, the “insufficient use of electronic records information on patients” decreased from 7.8 points in 2014 to 7.3 points in 2022, although it is not significant it reveals a trend toward an improvement during these years. The increased awareness of clinicians may in turn encourage regulatory institutions to set up guidelines and regulatory documents in the PGx field.

All of the above identified more relevant barriers in the LAC region overlap with barriers identified in other regions of the world, with some highlighted differences, especially in relation to developed countries (Jameson et al., 2021; Keeling et al., 2021; Virelli et al., 2021). Cost issues, lack of knowledge (patients and clinicians), trust in PGx results vendor and lack of clear guidelines, are barriers identified in many studies worldwide, with remarkable differences among developed, developing and undeveloped countries. In the case of LAC countries, as mainly developing countries cost issues are crucial to implement PGx in clinical practice and ethnicity questions are particularly relevant due to the great heterogeneity and the complex ethnic mixtures in the region (Homburger et al., 2015; Adhikari et al., 2016; Chacón-Duque et al., 2018; Norris et al., 2018; Statista, 2023; De Oliveira et al., 2023).

According to the survey results concerning barriers, the clinical demonstration and validation of PGx tests are key points for implementing PGx in LAC. Therefore, in the present survey, the inclusion of 54 gene/drug pairs was performed to evaluate concerns about the clinical utility of these PGx biomarkers (Figure 4). Forty-three pairs proposed by CPIC and eleven other pairs (non-CPIC) potentially relevant for the region were also evaluated. Data related to respondents (frequencies) were defined as the number of respondents who ranked the gene/drug pairs as 3, 4, or 5 among the total responses (on a scale of 1–5) plotted along the y-axis. We used this scale to make results comparable to our previous survey and others conducted on Spanish and US professionals (Relling and Klein, 2011; Agúndez et al., 2012; Quiñones et al., 2014). The 51 gene/drug pairs in the surveys performed in 2014 were included in the 2022 survey. However, after the clustering of some pairs by CPIC, as mentioned above, the total number of pairs appeared to not match among both surveys. Nevertheless, these are entirely comparable because the grouped pairs included drugs of the same family and similar metabolism. It is important to highlight that the level of relevance assigned by respondents to every pair in 2022 is considerably higher than in 2014, both for CPIC and non-CPIC pairs. This fact could reflect that LAC biomedical professionals are more knowledgeable about the use and application of gene/drug pairs, giving rise to the idea that LAC initiatives and educative activities are successful thus far. Our survey also has results similar to those obtained by Abou et al. (2019) with respect to the barriers for PGx implementation evaluated as challenges for bringing PGx into clinical practice. Their study was performed in 2014 with respondents from 43 countries (mainly from North America and Europe), and cost and ethics issues, skepticism of health professionals, unclear guidelines and test results, lack of adequate counselors who understand pharmacogenetic tests, inefficient administrative and regulatory bodies, extrapolation between populations and technical issues were identified as relevant challenges/barriers, giving rise to the idea that no large differences are observed among developed countries and Latin American countries, at least with respect to identified challenges.

According to the survey results, the highest ranked (96%–99%) gene/drug pairs perceived as important were *CYP2D6*/tamoxifen, *CYP3A5*/tacrolimus, *CYP2D6*/opioids, *DPYD*/fluoropyrimidines, *TMPT*/thiopurines, *CYP2D6*/tricyclic antidepressants, *CYP2C19*/tricyclic antidepressants, *NUDT15*/thiopurines, *CYP2B6*/efavirenz, and *CYP2C19*/clopidogrel; only in the *TMPT*/thiopurines for CPIC pairs was also found on the 2014 survey. Notably, all CPIC pairs were highly ranked by 77–99 respondents, with the lowest ranked pair being *COMT*/opioids (Figures 4A, B). With respect to the evaluated and comparable non-CPIC pairs, the highest ranked pairs (by 88–92 respondents) were *CYP2D6*/venlafaxine, *CYP2D6*/aripiprazole and *CYP2D6*/risperidone (Figures 4C, D), and all three had lower perceived importance in the 2014 survey (23, 29 and 23 respondents, respectively). The lowest ranked pair was *CYP2C19*/ethinyl-estradiol. Again, the non-CPIC pairs were also highly ranked by respondents (57–92) on the 2014 survey. This finding is likely a reflection of the improvement in the PGx scientific information available in our countries, giving rise to a better estimation of the importance of gene/drug pairs. The highest ranked gene/drug pairs in psychiatry and oncology suggest that LAC countries deal with similar health problems to developed countries.

Although the pairing of CYP2D6/SSRIs (selective serotonin reuptake inhibitors) was not one of the best-evaluated pairs, its importance has increased significantly from 15 in the 2014 survey to 85 in the 2022 survey (Figure 4A). This may be a positive signal of their growing clinical relevance in the region. This is significant since SSRIs, along with SNRIs (serotonin and norepinephrine reuptake inhibitors), are the first-line pharmacotherapy options for major depressive and anxiety disorders worldwide. The efficacy and potential side effects of these medications can vary depending on an individual’s CYP2D6 genotype, which may be highly variable in LAC countries (Perez-Paramo et al., 2015; Lochmann et al., 2017; Kane, 2021; Bousman et al., 2023).

Some limitations of this survey must be noted. First, even though the sample is considerably higher than in the 2014 survey and is probably more representative, it is nonetheless still small. Therefore, a future larger survey may be required for better results. However, the study participants from each country are people with at least basic knowledge in PGx. Second, the perceived importance of the gene/drug pairings could be different in each country of the region due to different access based on political, socioeconomic or administrative differences. Finally, the lack of sufficient cost–efficacy evidence of PGx, technical limitations in some countries and ethnic profiles of specific countries may significantly impact the implementation of PGx in clinical practice.

The implementation of technologies that involve target sequencing or in-house kits may represent a more affordable option for the region. In this respect, it is necessary to guarantee the identification of the most frequent genetic characteristics for these populations. However, once this main challenge is successfully overcome, the region will significantly benefit from precision medicine and public health. Collaborative work can provide meaningful information related to native and admixture PGx characteristics for each region, given the rich and variable response of LAC countries’ mixed populations.

## 5 Conclusion

Nowadays, even though the PGx capacity in LAC is increasing, this increase is at a similar rate to the global community, thus the contribution of Latin American and Caribbean countries to the PGx field worldwide remains limited. Our work shows improvement in both the number of publications as well as in the spread of knowledge and institutional efforts to address PGx as a relevant tool of personalized medicine in the region. Interestingly, the perception of the usefulness of PGx has drastically changed, raising awareness among physicians, which reflects a promising future in the clinical applications of PGx. The development of public policies and the implementation of intersectoral action plans that involve the different stakeholders of the health system continue to be a challenge in the implementation and growth of precision medicine in LAC.

## Data Availability

The raw data supporting the conclusion of this article will be made available by the authors, without undue reservation.
