# Supplementary material for: An updated examination of the perception of barriers for pharmacogenomics implementation and the usefulness of drug/gene pairs in Latin America and the Caribbean
Source: Front Pharmacol. 2023 May 11;14:1175737. doi: 10.3389/fphar.2023.1175737 (PMC10213898; doi:10.3389/fphar.2023.1175737)
Supplement: Supplementary file 1 [file DataSheet1.PDF]

Supplementary material

LAC SURVEY

Link: https://forms.gle/wnERH13ncFrD7yHj8

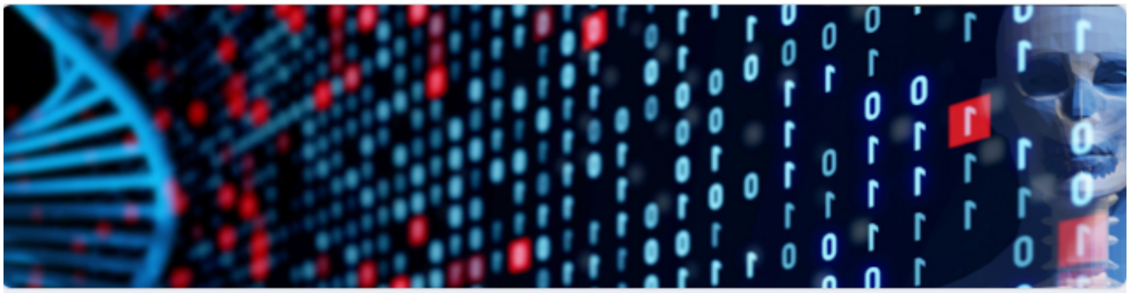

ENCUESTA: IMPLEMENTACIÓN FARMACOGENÓMICA EN AMERICA LATINA

Esta encuesta tiene por objeto evaluar los impedimentos, políticos, administrativos, sociales o clínicos existentes en la región Latinoamericana para la implementación de pruebas y protocolos farmacogenómicos, así mismo analizar que pares gen/fármaco consideran de relevancia para ser implementado en sus respectivos países.

edfarmaciaucr@gmail.com (no se comparten) Cambiar cuenta

*Obligatorio

Nombre *

Tu respuesta

País: *

Tu respuesta

Profesión: *

Tu respuesta

Correo electrónico: *

Tu respuesta

A. Considerando la relevancia la implementación de guías y ensayos farmacogenéticos/farmacogenómicos en Latinoamérica categorice en escala de 1 (baja relevancia) a 10 (alta relevancia), la relevancia de las siguientes barreras como impedimentos para implementación de la farmacogenómica en el ejercicio clínico.

1. Reticencia de los clínicos a usar marcadores farmacogenéticos *

	1	2	3	4	5	6	7	8	9	10	
Baja relevancia	○	○	○	○	○	○	○	○	○	○	Alta relevancia

2. Falta de clara información acerca de variantes genéticas que tengan relevancia funcional en la farmacoterapia *

	1	2	3	4	5	6	7	8	9	10	
Baja relevancia	○	○	○	○	○	○	○	○	○	○	Alta relevancia

3. Ausencia de instituciones o normas regulatorias que faciliten el uso de pruebas farmacogenéticas/farmacogenómicas *

	1	2	3	4	5	6	7	8	9	10	
Baja relevancia	○	○	○	○	○	○	○	○	○	○	Alta relevancia

4. Insuficiente caracterización de la variabilidad farmacogenética en Latinoamérica *

	1	2	3	4	5	6	7	8	9	10	
Baja relevancia	○	○	○	○	○	○	○	○	○	○	Alta relevancia

5. Insuficiente uso de medios electrónicos para obtener información de pacientes *

	1	2	3	4	5	6	7	8	9	10	
Baja relevancia	○	○	○	○	○	○	○	○	○	○	Alta relevancia

6. Falta de promoción de la farmacogenómica en los sistemas de salud *

	1	2	3	4	5	6	7	8	9	10	
Baja relevancia	○	○	○	○	○	○	○	○	○	○	Alta relevancia

7. Insuficiente preocupación de los clínicos en la farmacogenómica *

	1	2	3	4	5	6	7	8	9	10	
Baja relevancia	○	○	○	○	○	○	○	○	○	○	Alta relevancia

8. Necesidad de guías, procesos y protocolos de aplicación clínica de la farmacogenética/farmacogenómica. *

	1	2	3	4	5	6	7	8	9	10	
Baja relevancia	○	○	○	○	○	○	○	○	○	○	Alta relevancia

9. Necesidad de demostración de la validez clínica y utilidad de los test farmacogenéticos/farmacogenómicos *

	1	2	3	4	5	6	7	8	9	10	
Baja relevancia	○	○	○	○	○	○	○	○	○	○	Alta relevancia

10. Necesidad de implementación de ensayos gene/fármaco *

	1	2	3	4	5	6	7	8	9	10	
Baja relevancia	○	○	○	○	○	○	○	○	○	○	Alta relevancia

11. Preocupación acerca de los costos *

	1	2	3	4	5	6	7	8	9	10	
Baja relevancia	○	○	○	○	○	○	○	○	○	○	Alta relevancia

12. Falta de estudios farmacogenómicos de costo-efectividad en la región *

	1	2	3	4	5	6	7	8	9	10	
Baja relevancia	○	○	○	○	○	○	○	○	○	○	Alta relevancia

13. Fragmentación de los sistemas de salud *

	1	2	3	4	5	6	7	8	9	10	
Baja relevancia	○	○	○	○	○	○	○	○	○	○	Alta relevancia

14. Implicancias éticas, legales y sociales para la implementación de la farmacogenómica *

	1	2	3	4	5	6	7	8	9	10	
Baja relevancia	○	○	○	○	○	○	○	○	○	○	Alta relevancia

B. Considerando la relevancia del uso de los siguientes fármacos y la evidencia existente que usted conozca, categorice en escala de 1 a 5 la importancia de la implementación de determinaciones farmacogenómicas (variantes) de los genes involucrados. Considere que el listado se encuentra en el orden GEN-Fármaco(s). Para su apoyo se le adjunta links de acceso a CPIC y PharmGKB.
(https://cpicpgx.org/guidelines/ ; https://www.pharmgkb.org)

Categorice según las siguientes opciones: 1 (Muy poca importancia), 2 (Poca importancia), 3 (Moderada importancia), 4 (Importante) y 5 (Muy importante) *

	1	2	3	4	5
1. CFTR-Ivacaftor	○	○	○	○	○
2. CYP2B6-Efavirenz	○	○	○	○	○
3. CYP2C19-Clopidogrel	○	○	○	○	○
4. CYP2C19-Inhibidores de bomba de protones (prazoles)	○	○	○	○	○
5. CYP2C19-voriconazol	○	○	○	○	○
6. CYP2C9-Anti-inflamatorios no esteroidales (NSAIDs)	○	○	○	○	○
7. CYP2C9-fenitoína/fosfenitoína	○	○	○	○	○
8. HLA-B-fenitoína/fosfenitoína	○	○	○	○	○

9. CYP2C9-Cumarinas (warfarina, acenocumarol, fenprocumon)	○	○	○	○	○
10. VKORC1-Cumarinas (warfarina, acenocumarol, fenprocumon)	○	○	○	○	○
11. CYP4F2-Cumarinas (warfarina, acenocumarol, fenprocumon)	○	○	○	○	○
12. CYP2D6-Atomoxetina	○	○	○	○	○
13. CYP2D6-Ondasentrón/topisentrón	○	○	○	○	○
14. CYP2D6-tamoxifeno	○	○	○	○	○
15. CYP2D6- Inhibidores de la recaptación de Serotonina (citalopram, escitalopram)	○	○	○	○	○
16. CYP2C19- Inhibidores de la recaptación de Serotonina (fluvoxamina, paroxetina, sertralina)	○	○	○	○	○
17. CYP2D6- Antidepresivos tricíclicos (amitriptilina, clomipramina, desipramina)	○	○	○	○	○
18. CYP2C19- Antidepresivos tricíclicos (doxepina, imipramina, nortriptilina, trimipramina).	○	○	○	○	○
19. CYP2D6-Opioides	○	○	○	○	○

20. OPRM1-Opioides					
21. COMT-Opioides					
22. CYP3A5-Tacrolimus					
23. DPYD-Fluoropirimidinas (5-Fluorouracilo, capecitabina, tegafur)					
24. G6PD-rasburicasa					
25. HLA-Carbamazepina/oxcarbazepina					
26. HLA-B-Abacavir					
27. HLA-B-Alopurinol					
28. IFNL3-peginterferón					
29. MT-RNR1-aminoglicósidos					
30. RYR1-Enflurano o derivados					
31. CACNA1S- Enflurano o derivados					
32. SLCO1B1-Estatinas					
33. ABCG2-Estatinas					

34. CYP2C9-Estatinas	○	○	○	○	○
35. TPMT-Tiopurinas (azatioprina, mercaptopurinas, tioguanina)	○	○	○	○	○
36. NUDT15- Tiopurinas (azatioprina, mercaptopurinas, tioguanina)	○	○	○	○	○
37. UGT1A1-Atazanavir	○	○	○	○	○

PARES NO-CPIC *

	1	2	3	4	5
1. CYP2C19-Drospirenona	○	○	○	○	○
2. CYP2C19-Etinilestradiol	○	○	○	○	○
3. CYP3A4-Benzodiazepinas	○	○	○	○	○
4. CYP2D6-Benzodiazepinas	○	○	○	○	○
5. CYP2D6-aripiprazol	○	○	○	○	○
6. CYP2D6-carvedilol	○	○	○	○	○

7. CYP2D6-venlafaxina	○	○	○	○	○
8. CYP2D6-risperidona	○	○	○	○	○
9. CYP1A2-clozapina/olanzapina	○	○	○	○	○
10. CYP3A4-Haloperidol	○	○	○	○	○
11. GSTs-cisplatino	○	○	○	○	○

Mencione algún otro par gen-droga que usted considere relevante, donde exista evidencia científica.

Tu respuesta

Enviar

Borrar formulario

Nunca envíes contraseñas a través de Formularios de Google.

Google no creó ni aprobó este contenido. Denunciar abuso - Condiciones del Servicio - Política de Privacidad

Google Formularios

PGX WORLDWIDE PUBLICATIONS (1959-2022)

Year	Number of Publicacions	
2022	1521	
2021	1762	
2020	1710	
2019	1674	
2018	1758	
2017	1848	
2016	1870	
2015	1900	
2014	1997	
2013	2066	
2012	2119	
2011	1917	
2010	1997	
2009	1807	
2008	1933	
2007	1644	
2006	1554	
2005	1425	
2004	1142	
2003	1015	
2002	838	
2001	625	
2000	430	
1999	251	
1998	141	
1997	94	
1996	76	
1995	78	
1994	54	
1993	66	
1992	63	
1991	54	
1990	55	
1989	70	
1988	45	
1987	74	
1986	95	
1985	63	
1984	50	
1983	66	
1982	60	
1981	39	
1980	50	
1979	64	
1978	74	
1977	49	
1976	46	
1975	57	
1974	75	
1973	59	
1972	33	
1971	44	
1970	41	
1969	30	
1968	50	
1967	29	
1966	9	
1965	8	
1964	13	
1963	4	
1962	2	
1961	1	
1960	1	
1959	1	
	38786	38786

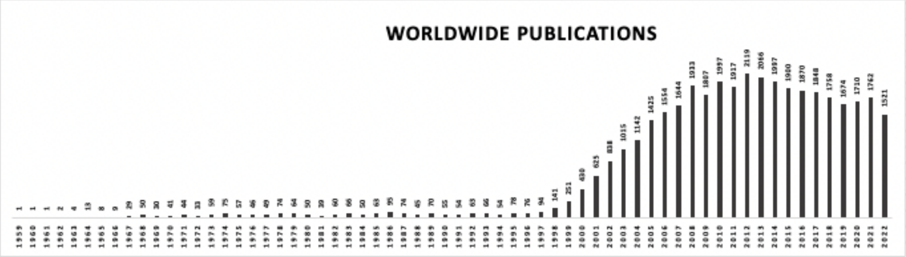

<u>**PGX-RELATED CLINICAL TRIALS WORLDWIDE (1996-2022)**</u>

Year	Trials
2022	30
2021	31
2020	36
2019	34
2018	29
2017	45
2016	37
2015	45
2014	40
2013	34
2012	41
2011	47
2010	46
2009	49
2008	47
2007	44
2006	44
2005	38
2004	21
2003	15
2002	7
2001	3
2000	2
1999	6
1998	1
1997	1
1996	1

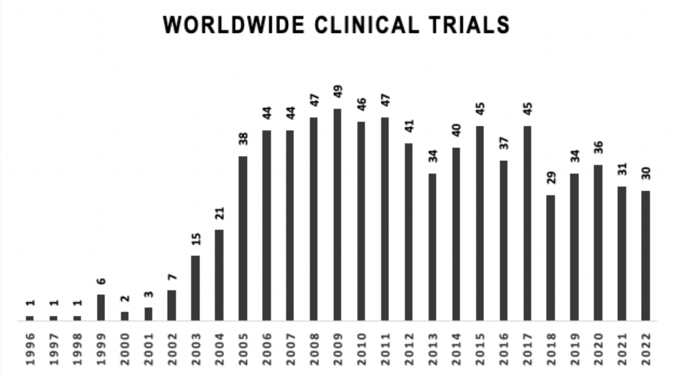

PGX LAC PUBLICATIONS BY COUNTRY

Países	Número de Publicaciones	Responden la encuesta	%
BRAZIL	561	SI	41,95961107
MEXICO	231	SI	17,27748691
CHILE	120	SI	8,975317876
ARGENTINA	104	SI	7,778608826
COLOMBIA	91	SI	6,806282723
PUERTO RICO	43	NO	3,216155572
PERU	31	SI	2,318623785
CUBA	33	SI	2,468212416
ECUADOR	30	SI	2,243829469
URUGUAY	25	SI	1,869857891
VENEZUELA	20	SI	1,495886313
COSTA RICA	16	SI	1,19670905
PANAMA	10	NO	0,747943156
NICARAGUA	9	SI	0,673148841
GUATEMALA	5	SI	0,373971578
JAMAICA	2	NO	0,149588631
HAITI	3	NO	0,224382947
BOLIVIA	1	SI	0,074794316
HONDURAS	1	SI	0,074794316
EL SALVADOR	1	SI	0,074794316
BÉLICE	0	SI	
	1337		

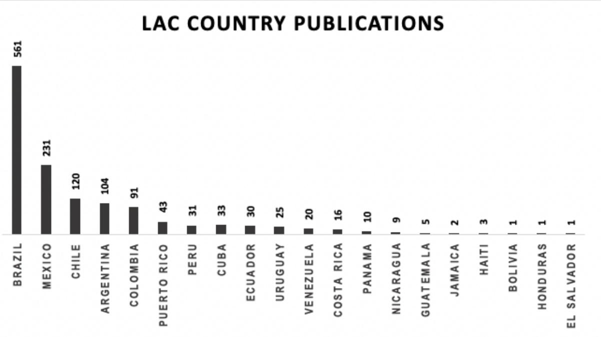

PGX LAC PUBLICATIONS BY YEAR (1984-2022)

Año	Número de Publicaciones
2022	74
2021	80
2020	90
2019	81
2018	86
2017	81
2016	72
2015	68
2014	77
2013	56
2012	65
2011	51
2010	54
2009	39
2008	52
2007	36
2006	26
2005	18
2004	22
2003	17
2002	6
2001	5
2000	1
1999	5
1998	1
1997	0
1996	0
1995	1
1994	1
1993	1
1992	0
1991	0
1990	0
1989	0
1988	1
1987	0
1986	0
1985	0
1984	1

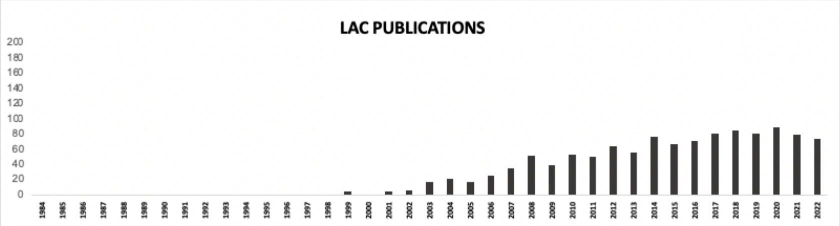

LAC CLINICAL TRIALS (2003-2022)

Clinical trials LAC				
2022	0			
2021	0			
2020	2		CHILE, MÉXICO, PERÚ	MÉXICO
2019	1		BRASIL	
2018	1		MÉXICO, PERÚ	
2017	2	COLOMBIA	CHILE, MÉXICO, PERÚ	
2016	1		MÉXICO	
2015	1		URUGUAY	
2014	1	PUERTO RICO		
2013	2		BRASIL	PUERTO RICO
2012	0			
2011	0			
2010	1		BRAZIL	
2009	1	CHILE		
2008	0			
2007	1		ARGENTINA	
2006	2	PUERTO RICO	ARGENTINA	
2005	2	MÉXICO	PUERTO RICO	
2004	0			
2003	1		MÉXICO	
2002	0			
2001	0			
2000	0			
1999	0			
1998	0			
1997	0			
1996	0			
	19			

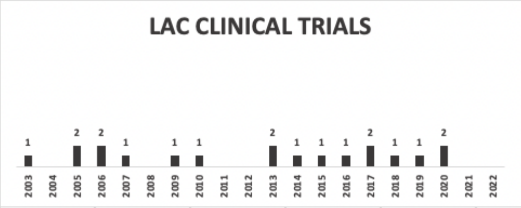

LAC PGx Courses: Links:

1. https://www.fq.edu.uy/?q=es/node/1269
2. https://www.favaloro.edu.ar/informacion/bioFARG_curso-de-farmacogenomica/
3. https://www.fcq.unc.edu.ar/node/128
4. https://www.infobioquimica.com/new/2020/08/12/entrevista-con-el-dr-german-perez-argentina-curso-virtual-de-farmacogenomica-en-oncologia/
5. https://www.portaleducacao.com.br/curso-online-farmacia-farmacogenetica/p?utm_term=&utm_campaign=2022_abr_mai_conv-purch_vds_portal-educacao_play-estudante-ongoing&utm_source=adwords&utm_medium=ppc&hsa_acc=2284317825&hsa_cam=17076386131&hsa_grp=&hsa_ad=&hsa_src=x&hsa_tgt=&hsa_kw=&hsa_mt=&hsa_net=adwords&hsa_ver=3&gclid=CjwKCAjwiOCgBhAgEiwAjv5whNfjrf-_qXQghcc1TrIJ-Np3mJVFMiumgpd8gO4YiHOAABSGQfVgnhoC53MQAvD_BwE
6. https://www.galileo.edu/fabiq/carrera/maestria-biologia-molecular/pensum/
7. https://cursalud.cl/producto/curso-de-farmacogenetica-y-farmacogenomica-e-learning/
8. https://www.uv.mx/mlcat/files/2019/06/Farmacogenomica.pdf
9. https://www.inmegen.gob.mx/media/filer_public/d6/82/d68282c4-28f7-4fe1-aa70-e38e5c45077d/33_edc_farma_facebook.jpg

STATISTICS

		Average (2014)	Standard deviation + -(2014)		Average (2022)	Standard deviation + - (2022)	*p- value **
Reluctance of clinicians to use pharmacogenetic markers	20	6,748466258	1,355828221	106	5,575471698	2,522085732	***0,0042***
Lack of clear information about genetic variants that will have functional relevance in pharmacotherapy	20	7,141104294	1,116564417	106	7,056603774	2,731928287	*0,8173*
Absence of institutions or regulatory norms that facilitate the use of pharmacogenetic/pharmacogenomic tests	20	8,45398773	1,18404908	106	8,849056604	1,845418795	*0,2240*
Insufficient characterization of pharmacogenetic variability in Latin American populations	20	8,141104294	1,153374233	106	8,075471698	1,993790901	*0,8397*
Insufficient use of electronic records information on patient	20	7,797546012	1,030674847	106	7,311320755	2,485628052	*0,1497*
Healthcare systems do not promote pharmacogenomics use	20	7,748466258	1,90797546	106	8,858490566	2,063001416	***0,0257***
Insufficient concern about pharmacogenomics among clinicians	20	8,496932515	0,932515337	106	7,301886792	2,403052865	***0,0003***
Need for clear guidelines, processes and protocols for the clinical application of pharmacogenetics/pharmacogenomics in LAC	20	8,748466258	0,914110429	106	9,056603774	1,73934957	*0,2508*
Need for demonstration of clinical validity and utility of pharmacogenetic/pharmacogenomic tests	20	6,889570552	1,122699387	106	8,556603774	1,867415778	***<0,0001***
Need for implementation of gene/drug trials	20	7,423312883	0,993865031	106	8,745283019	1,621647139	***<0,0001***
Concerns about test costs	20	7,484662577	1,104294479	106	8,481132075	1,837343486	***0,0021***
Lack of cost-effectiveness pharmacogenomics studies in the region	20	**	**	106	8,962264151	1,5487293	
Fragmentation of healthcare systems	20	8,325153374	1,073619632	106	8,103773585	2,284228515	*0,5010*
Ethical, legal and social implications for the implementation of pharmacogenomics	20	6,049079755	1,214723926	106	7,58490566	2,49138678	***0,0001***
		**N (year 2014) = 20**			**N (year 2022) = 106**		

*** Two-sample student test with unequal variances**

* Empty data: No requested in 2014

Reluctance of clinicians to use pharmacogenetic markers

Two-sample t test with unequal variances

	Obs	Mean	Std. err.	Std. dev.	[95% conf. interval]	
x	20	6,04908	,2716205	1,214724	5,480571	6,617588
y	106	7,584906	,2419849	2,491387	7,105094	8,064717
Combined	126	7,341124	,2137286	2,399097	6,918128	7,764119
diff		-1,535826	,3637779		-2,264891	-,806761

diff = mean(x) - mean(y) t = -4,2219
H0: diff = 0 Satterthwaite's degrees of freedom = 54,8741

Ha: diff < 0 Pr(T < t) = 0,0000
Ha: diff != 0 Pr(|T| > |t|) = 0,0001
Ha: diff > 0 Pr(T > t) = 1,0000

. ttesti 20 6.748466258 1.355828221 106 5.575471698 2.522085732, une

Lack of clear information about genetic variants that will have functional relevance in pharmacotherapy

Two-sample t test with unequal variances

	Obs	Mean	Std. err.	Std. dev.	[95% conf. interval]	
x	20	6,748466	,3031724	1,355828	6,113919	7,383013
y	106	5,575472	,2449666	2,522086	5,089748	6,061195
Combined	126	5,761661	,2146941	2,409936	5,336755	6,186568
diff		1,172995	,3897719		,3893859	1,956603

diff = mean(x) - mean(y) t = 3,0094
H0: diff = 0 Satterthwaite's degrees of freedom = 48,1913

Ha: diff < 0 Pr(T < t) = 0,9979
Ha: diff != 0 Pr(|T| > |t|) = 0,0042
Ha: diff > 0 Pr(T > t) = 0,0021

. ttesti 20 7.141104294 1.116564417106 106 7.056603774 2.731928287, une

Absence of institutions or regulatory norms that facilitate the use of pharmacogenetic/pharmacogenomic tests

Two-sample t test with unequal variances

	Obs	Mean	Std. err.	Std. dev.	[95% conf. interval]	
x	20	7,141104	,2496714	1,116564	6,618536	7,663673
y	106	7,056604	,2653483	2,731928	6,530467	7,58274
Combined	126	7,070017	,226424	2,541603	6,621895	7,518138
diff		,0845005	,3643426		-,6421576	,8111586

diff = mean(x) - mean(y) t = 0,2319
H0: diff = 0 Satterthwaite's degrees of freedom = 70,0017

Ha: diff < 0 Pr(T < t) = 0,5914
Ha: diff != 0 Pr(|T| > |t|) = 0,8173
Ha: diff > 0 Pr(T > t) = 0,4086

. ttesti 20 8.45398773 1.18404908 106 8.849056604 1.845418795, une

Insufficient characterization of pharmacogenetic variability in Latin American populations

```
Two-sample t test with unequal variances

             Obs       Mean    Std. err.   Std. dev.   [95% conf. interval]

       x      20   8,453988    ,2647614    1,184049    7,899836     9,00814
       y     106   8,849057    ,1792429    1,845419    8,493651    9,204462

Combined     126   8,786347    ,1567221    1,759201    8,476175     9,09652

    diff          -,3950689     ,319729                -1,04182    ,2516824

    diff = mean(x) - mean(y)                                      t =  -1,2356
H0: diff = 0                     Satterthwaite's degrees of freedom =  38,9279

    Ha: diff < 0                 Ha: diff != 0                 Ha: diff > 0
 Pr(T < t) = 0,1120         Pr(|T| > |t|) = 0,2240          Pr(T > t) = 0,8880

. ttesti 20 8.141104294 1.153374233 106 8.075471698 1.993790901, une
```

Insufficient use of electronic records information on patient

```
Two-sample t test with unequal variances

             Obs       Mean    Std. err.   Std. dev.   [95% conf. interval]

       x      20   8,141104    ,2579023    1,153374    7,601309      8,6809
       y     106   8,075472    ,1936541    1,993791    7,691491    8,459452

Combined     126    8,08589    ,1676625    1,882007    7,754065    8,417715

    diff           ,0656326    ,3225144               -,5843784    ,7156436

    diff = mean(x) - mean(y)                                      t =   0,2035
H0: diff = 0                     Satterthwaite's degrees of freedom =  43,9378

    Ha: diff < 0                 Ha: diff != 0                 Ha: diff > 0
 Pr(T < t) = 0,5802         Pr(|T| > |t|) = 0,8397          Pr(T > t) = 0,4198

. ttesti 20 7.797546012 1.030674847 106 7.311320755 2.485628052, une
```

Healthcare systems do not promote pharmacogenomics use

```
Two-sample t test with unequal variances

             Obs       Mean    Std. err.   Std. dev.   [95% conf. interval]

       x      20   7,797546    ,2304659    1,030675    7,315175    8,279917
       y     106   7,311321    ,2414255    2,485628    6,832619    7,790023

Combined     126   7,388499    ,2066955    2,320151    6,979423    7,797575

    diff           ,4862253    ,3337676               -,1796876    1,152138

    diff = mean(x) - mean(y)                                      t =   1,4568
H0: diff = 0                     Satterthwaite's degrees of freedom =  68,6262

    Ha: diff < 0                 Ha: diff != 0                 Ha: diff > 0
 Pr(T < t) = 0,9251         Pr(|T| > |t|) = 0,1497          Pr(T > t) = 0,0749

. ttesti 20 7.748466258 1.90797546 106 8.858490566 2.063001416, une
```

Insufficient concern about pharmacogenomics among clinicians

Two-sample t test with unequal variances

	Obs	Mean	Std. err.	Std. dev.	[95% conf.	interval]
x	20	7,748466	,4266363	1,907975	6,855506	8,641426
y	106	8,858491	,2003764	2,063001	8,461181	9,2558
Combined	126	8,682296	,1846104	2,072247	8,316929	9,047663
diff		-1,110024	,4713483		-2,075445	-,1446036

diff = mean(x) - mean(y)		t = -2,3550
H0: diff = 0		Satterthwaite's degrees of freedom = 28,0597
Ha: diff < 0	Ha: diff != 0	Ha: diff > 0
Pr(T < t) = 0,0129	Pr(|T| > |t|) = 0,0257	Pr(T > t) = 0,9871

. ttesti 20 8.496932515 0.932515337 106 7.301886792 2.403052865, une

Need for clear guidelines, processes and protocols for the clinical application of pharmacogenetics/pharmacogenomics in LAC

Two-sample t test with unequal variances

	Obs	Mean	Std. err.	Std. dev.	[95% conf.	interval]
x	20	8,496933	,2085168	,9325153	8,060502	8,933363
y	106	7,301887	,2334051	2,403053	6,839088	7,764686
Combined	126	7,491577	,2026633	2,27489	7,090481	7,892672
diff		1,195046	,3129811		,5715696	1,818522

diff = mean(x) - mean(y)		t = 3,8183
H0: diff = 0		Satterthwaite's degrees of freedom = 75,1052
Ha: diff < 0	Ha: diff != 0	Ha: diff > 0
Pr(T < t) = 0,9999	Pr(|T| > |t|) = 0,0003	Pr(T > t) = 0,0001

. ttesti 20 8.748466258 0.914110429 106 9.056603774 1.73934957, une

Need for demonstration of clinical validity and utility of pharmacogenetic/pharmacogenomic tests

Two-sample t test with unequal variances

	Obs	Mean	Std. err.	Std. dev.	[95% conf.	interval]
x	20	8,748466	,2044013	,9141104	8,320649	9,176283
y	106	9,056604	,1689406	1,73935	8,721626	9,391582
Combined	126	9,007693	,1458711	1,637399	8,718996	9,29639
diff		-,3081375	,2651807		-,840866	,2245909

diff = mean(x) - mean(y)		t = -1,1620
H0: diff = 0		Satterthwaite's degrees of freedom = 49,6341
Ha: diff < 0	Ha: diff != 0	Ha: diff > 0
Pr(T < t) = 0,1254	Pr(|T| > |t|) = 0,2508	Pr(T > t) = 0,8746

. ttesti 20 6.889570552 1.122699387 106 8.556603774 1.867415778, une

Need for implementation of gene/drug trials

Two-sample t test with unequal variances

	Obs	Mean	Std. err.	Std. dev.	[95% conf.	interval]
x	20	6,889571	,2510432	1,122699	6,364131	7,41501
y	106	8,556604	,1813795	1,867416	8,196962	8,916246
Combined	126	8,291995	,166546	1,869474	7,96238	8,621611
diff		-1,667033	,3097115		-2,29208	-1,041986

diff = mean(x) - mean(y) t = -5,3825

H0: diff = 0 Satterthwaite's degrees of freedom = 41,9455

Ha: diff < 0 Pr(T < t) = 0,0000

Ha: diff != 0 Pr(|T| > |t|) = 0,0000

Ha: diff > 0 Pr(T > t) = 1,0000

. ttesti 20 7.423312883 0.993865031 106 8.745283019 1.621647139, une

Concerns about test costs

Two-sample t test with unequal variances

	Obs	Mean	Std. err.	Std. dev.	[95% conf.	interval]
x	20	7,423313	,222235	,993865	6,95817	7,888456
y	106	8,745283	,1575083	1,621647	8,432973	9,057593
Combined	126	8,535446	,1434926	1,6107	8,251457	8,819436
diff		-1,32197	,2723917		-1,872072	-,771868

diff = mean(x) - mean(y) t = -4,8532

H0: diff = 0 Satterthwaite's degrees of freedom = 41,01

Ha: diff < 0 Pr(T < t) = 0,0000

Ha: diff != 0 Pr(|T| > |t|) = 0,0000

Ha: diff > 0 Pr(T > t) = 1,0000

. ttesti 20 7.484662577 1.104294479 106 8.481132075 1.837343486, une

Fragmentation of healthcare systems

Two-sample t test with unequal variances

	Obs	Mean	Std. err.	Std. dev.	[95% conf.	interval]
x	20	7,484663	,2469278	1,104294	6,967837	8,001488
y	106	8,481132	,1784586	1,837343	8,127282	8,834982
Combined	126	8,322962	,1582321	1,776151	8,009801	8,636123
diff		-,9964695	,304665		-1,611326	-,3816133

diff = mean(x) - mean(y) t = -3,2707

H0: diff = 0 Satterthwaite's degrees of freedom = 41,9602

Ha: diff < 0 Pr(T < t) = 0,0011

Ha: diff != 0 Pr(|T| > |t|) = 0,0021

Ha: diff > 0 Pr(T > t) = 0,9989

. ttesti 20 8.325153374 1.073619632 106 8.103773585 2.284228515, une

Two-sample t test with unequal variances

	Obs	Mean	Std. err.	Std. dev.	[95% conf.	interval]
x	20	8,325153	,2400686	1,07362	7,822684	8,827623
y	106	8,103774	,2218639	2,284229	7,663858	8,543689
Combined	126	8,138913	,1903353	2,136509	7,762216	8,51561
diff		,2213798	,3268892		-,4330327	,8757923

diff = mean(x) - mean(y) t = 0,6772
H0: diff = 0 Satterthwaite's degrees of freedom = 57,699

Ha: diff < 0 Ha: diff != 0 Ha: diff > 0
Pr(T < t) = 0,7495 Pr(|T| > |t|) = 0,5010 Pr(T > t) = 0,2505

. ttesti 20 6.049079755 1.214723926 106 7.58490566 2.49138678, une
